# Identification and Analysis of NBS-LRR Genes in *Actinidia chinensis* Genome

**DOI:** 10.3390/plants9101350

**Published:** 2020-10-13

**Authors:** Tao Wang, Zhan-Hui Jia, Ji-Yu Zhang, Min Liu, Zhong-Ren Guo, Gang Wang

**Affiliations:** Institute of Botany, Jiangsu Province and Chinese Academy of Sciences, Nanjing 210014, China; wangtao@cnbg.net (T.W.); jiazhanhui@cnbg.net (Z.-H.J.); maxzhangjy@cnbg.net (J.-Y.Z.); liumin@cnbg.net (M.L.); zhongrenguo@cnbg.net (Z.-R.G.)

**Keywords:** kiwifruit, resistance, R-genes, kiwifruit bacterial canker

## Abstract

Nucleotide-binding site and leucine-rich repeat (NBS-LRR) genes represent the most important disease resistance genes in plants. The genome sequence of kiwifruit (*Actinidia chinensis*) provides resources for the characterization of NBS-LRR genes and identification of new R-genes in kiwifruit. In the present study, we identified 100 NBS-LRR genes in the kiwifruit genome and they were grouped into six distinct classes based on their domain architecture. Of the 100 genes, 79 are truncated non-regular NBS-LRR genes. Except for 37 NBS-LRR genes with no location information, the remaining 63 genes are distributed unevenly across 18 kiwifruit chromosomes and 38.01% of them are present in clusters. Seventeen families of cis-acting elements were identified in the promoters of the NBS-LRR genes, including AP2, NAC, ERF and MYB. *Pseudomonas syringae* pv. *actinidiae* (pathogen of the kiwifruit bacterial canker) infection induced differential expressions of 16 detected NBS-LRR genes and three of them are involved in plant immunity responses. Our study provides insight of the NBS-LRR genes in kiwifruit and a resource for the identification of new R-genes in the fruit.

## 1. Introduction

Kiwifruit (*Actinidia chinensis*) is well known for its high vitamin C content and spectacular flavor and it is now popular with more and more people. Particularly, the new varieties with red fresh for example ‘Hongyang’ which is derived from *A. chinensis* var. *chinensis* [1] are loved by a lot of Chinese people. With the rapid growth of the planting area, various diseases bring a serious threat to kiwifruit. One of the most serious diseases is the kiwifruit bacterial canker, which once brought about serious losses, especially to New Zealand. From the year 2010 to 2012, 37% of orchards in New Zealand were infected by bacterial canker [2]. As more and more countries grow kiwifruit and due to the strong transmission capacity of the pathogen, now the disease has spread to the main kiwifruit planting countries, such as Portugal [3], Spain [4], France [5], Turkey [6], Greece [7] and China.

Encoding products of most disease resistance genes in plants are nucleotide-binding site leucine-rich repeat (NBS-LRR) proteins and diverse pathogens, including bacteria, viruses, fungi, nematodes, insects and oomycetes, are detected by NBS-LRR proteins. Researchers have studied this gene family in many plant genomes, including *Arabidopsis thaliana* [8], *Glycine max* [9], *Lotus japonicas* [10], *Helianthus annuus* [11], *Oryza sativa* [12] and *Triticum aestivum* [13]. Due to the difference of the N-terminal features, the NBS-LRR proteins can be divided into two subfamilies, TIR-NBS-LRR (TNL) and non-TNL-NBS-LRR (nTNL) [14]. TNLs carry the *Drosophila* Toll and Interleukin-1 receptor (TIR) domain and nTNLs have the coil–coil (CC) domain at the N-terminal position. The TIR domain and CC domain were demonstrated to function in pathogen recognition. The NBS domain is highly conserved and it plays important role in signal transduction [15,16,17,18,19]. It was also found to function in resistance specificity [20,21,22,23]. The LRR motif is typically involved in protein–protein interactions and is responsible for recognition specificity [24]. With the rapid development of molecular biology and bioinformatics, the mining and positioning of functional genes in plant genome-wide data have become research hotspots. The released whole genome sequences of *A. chinensis* allowed for systematic analysis of NBS-LRR genes in kiwifruit. In this study, we identified NBS-LRR genes from *A. chinensis* genome using a bioinformatics approach. The chromosomal location, gene clusters, and phylogenetic relationships of these genes were analyzed. The expression of NBS-LRR genes in *A. chinensis* was analyzed after *Pseudomonas syringae* pv. *actinidiae* (Psa) infection.

## 2. Results

### 2.1. Identification and Classification of Kiwifruit NBS-LRR Genes

In total, 100 NBS-LRR protein coding sequences were identified in *A. chinensis* (Table S1). Based on the protein domain combinations, these genes were further grouped into six classes (Table 1). All the 100 genes contain the NBS domain and 20 only contain this one domain. In summary, all of the NBS-LRR genes contain the NBS domain, 76% contain the LRR domain, 23% contain the CC domain and only 2% contain the TIR domain. Usually, these domains are evenly distributed in the NBS-LRR genes, but there are some exceptions. Four CC domains were detected in the CC-NBS-LRR-type gene *Achn337321* and two were detected in *Achn334471* and *Achn096341*. Two NBS domains were detected in NBS-LRR-type genes *Achn388651* and *Achn348041*.

### 2.2. Phylogenetic Analysis, Gene Structural Characteristics, and Chromosome Localization

**Figure 1:**
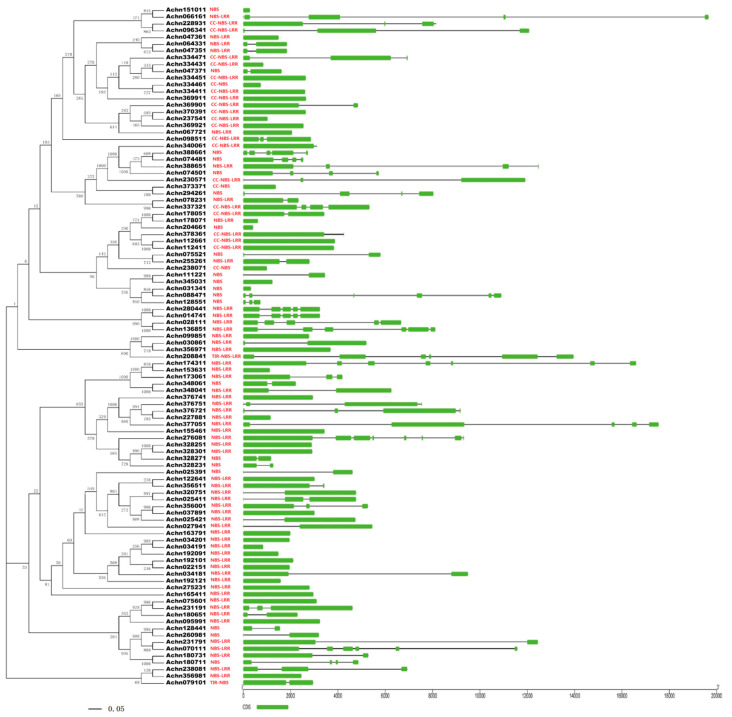
Phylogenetic tree and exon/intron structure of NBS-LRR genes of kiwifruit. The phylogenetic tree was generated using MAGA 5.0 with neighbor joining (NJ) method based on 1000 bootstrap replicates. The gene structure was retrieved from the General Feature File of kiwifruit genome. The phylogenetic tree was aligned with the gene structure along with the domain position using Gene Structure Display Serve.

**Figure 2:**
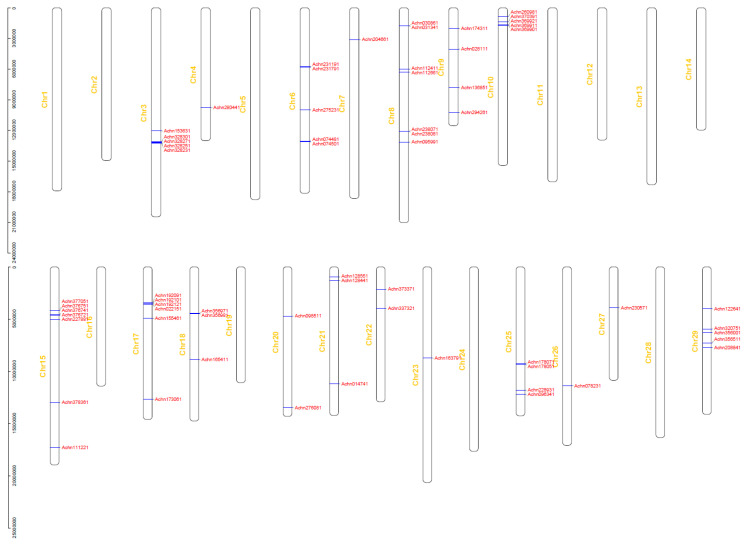
Chromosomal distribution and gene clusters of the NBS-LRR genes of kiwifruit.

A phylogenetic tree (Figure 1) was constructed based on protein sequences of the 100 NBS-LRR proteins using the neighbor-joining (NJ) method. The 20 CC-NBS-LRR-type genes were clearly clustered and separated from the TIR-NBS-LRR-type gene and TIR-NBS-type genes. Alignment of the exon/intron structure was conducted with the phylogenetic tree. The average length of coding sequences (CDSs) of the kiwifruit NBS-LRR genes is 2293 bp, which codes 763 amino acids. The average number of exons of the 100 NBS-LRR genes is 2.28. The NBS-LRR-type gene *Achn192121* contains the highest number (seven) of exons and 43 NBS-LRR genes contain only one exon. Some genes within the same cluster also displayed the same or a similar exon/intron structure and some genes with the same structure clustered together in pairs in the phylogenetic tree and they were also similar in sequence. Proteins Achn328251 and Achn328301 showed 96.09% identity in amino acid sequences, and sequences of proteins Achn064331 and Achn047351 were identical. The physical locations of the NBS-LRR genes were determined based on the kiwifruit gene annotation. Thirty-seven genes were removed due to a lack of chromosome location information, and the remaining 63 genes were detected on 18 chromosomes of the 29 kiwifruit chromosomes (Figure 2). These genes were not evenly distributed on the chromosomes. The most genes were located on chromosomes 8 and 15 which contained seven genes, respectively. Genes on the same chromosome tended to form clusters. Nine gene clusters were found on seven chromosomes. Two clusters contained four genes, two contained three genes and the remaining five clusters contained two genes. Genes within one cluster were usually similar in sequence and showed a closer evolutionary relationship than with other genes. This phenomenon was found in seven gene clusters of all the nine clusters. For example, genes *Achn328251*, *Achn328301*, *Achn328271* and *Achn328231*, which formed a gene cluster on chromosome 3, were also classified into one small group in the phylogenetic tree. Genes *Achn178051* and *Achn178071*, and genes *Achn112661* and *Achn112411,* which located closely but not in one gene cluster, respectively, were clustered in pairs in the phylogenetic tree as well.

### 2.3. Cis-Acting Element Analysis and Subcellular Location Prediction

**Figure 3:**
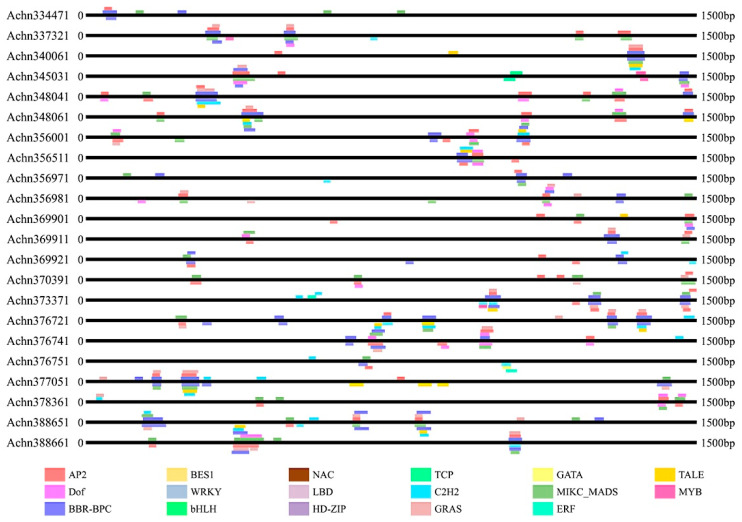
Cis-acting elements of promoters of 22 representative NBS-LRR genes. Cis-elements were predicted and analyzed using the PALCE database. Data of all the 100 NBS-LRR genes are displayed in Figure S1.

To investigate the potential functions and transcriptional regulation of these kiwifruit NBS-LRR genes, 1500 bp sequences upstream the start codon of the 100 NBS-LRR genes were isolated to identify putative cis-acting elements. In total, cis-acting elements belonging to 17 families (Figure 3 and Figure S1) were identified in the promoter regions of the kiwifruit NBS-LRR genes and they were involved in various life processes, including growth, development, stress response, hormones, signal transduction and other processes. Cis-acting elements were detected in every NBS-LRR gene and the gene with the fewest cis-acting elements had two. The NBS-LRR-type gene *Achn028111* was identified with as many as 39 cis-acting elements which belonged to seven families. Cis-acting elements were detected on both the sense strand and antisense strand and they seemed to be distributed randomly. These cis-acting elements were not distributed evenly. Many cis-acting elements tended to be clustered on the same locus or across a limited length of sequence. Some loci were identified with as many as eight cis-acting elements. For many genes, several cis-acting elements belonging to the same family were detected. Just as gene structure, the genes with a close genetic relationship, also had similar cis-elements, such as *Achn328251*, *Achn328301*, *Achn328271* and *Achn328231*. A large number of cis-elements involved in plant growth were detected, such as HD-Zip, TCP, MIKC-MADS and Dof. Furthermore, cis-elements associated with stress responses (AP2, NAC, ERF and MYB) were also identified in the kiwifruit NBS-LRR genes.

To analyze the possible location where these kiwifruit NBS-LRR proteins function, we predicted the subcellular locations of these proteins. Most of the kiwifruit NBS-LRR proteins were predicted to be located in multiple organelles (Table S2). The most possible organelles these proteins tend to locate in included chloroplasts, cytoskeleton, cytosol, endocytoplasmic reticulum, extracellular, mitochondrion, nuclei, peroxisome, plastid and vacuoles. Forty proteins were predicted to be located in the cytoskeleton and 38 in the nucleus.

### 2.4. Expression Pattern of Kiwifruit NBS-LRR Genes under Psa Infection

**Figure 4:**
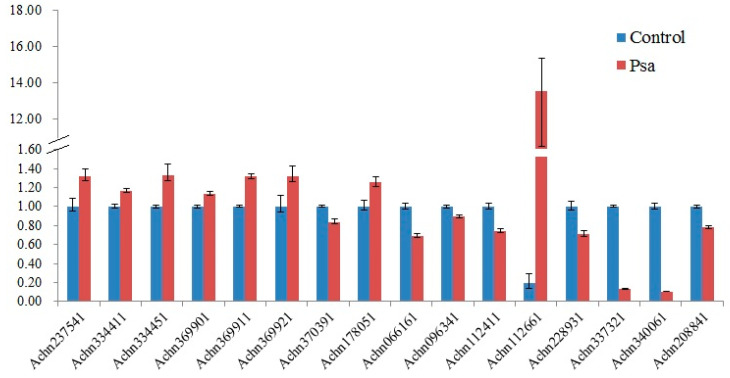
Expression level of 16 NBS-LRR genes. Kiwifruit cultivar “Hongyang” (*A. chinensis* var. *chinensis*) was used. Shoots were collected from 5 kiwifruit trees and stuck in MS medium. Psa was injected into the stems after they were carved with a knife. Phloem was collected after 48 h with three biological replicates.

The 20 CNL-type genes and one TNL-type gene were chosen for qRT-PCR profiling. At last, the expression of 16 NBS-LRR genes was successfully detected, because we cannot get suitable primers for the remaining five genes. All the 16 NBS-LRR genes were differentially expressed after Psa infection (Figure 4); eight were upregulated and the remaining eight were downregulated. Among the examined 16 genes, the expression of three genes was over the cut-off level of 2.0-fold mean change. Psa infection significantly induced the expression of the CNL-type gene *Achn112661* and the expression of *Achn337321* and *Achn340061* were significantly downregulated.

## 3. Discussion

As the planting area increased quickly, the major planting kiwifruit varieties lost their resistance to many kinds of diseases. Kiwifruit bacterial canker, which is the most serious disease in kiwifruit planting, swept the world in just a few years and led to a serious drop in production [25]. So, creating new resistant kiwifruit varieties is the primary task of kiwifruit breeders. As the genome of kiwifruit has been sequenced, it provides the chance to breed resistant varieties in the use of resistance genes. NBS-LRR genes are the most important resistance genes in plants and some members of them have been found to play a key role in the resistance to pathogens of *Pseudomonas syringae* [26,27]. Here, we characterize the complete set of NBS-LRR genes in kiwifruit genome, hoping to provide basic information for the breeding of new resistant kiwifruit varieties.

In the genome of kiwifruit, the identified NBS-LRR genes represented about 0.256% of all the predicted kiwifruit open reading frames (ORFs). This number was higher than that of papaya (0.145%) and cucumber (0.0821%), and smaller than Arabidopsis (0.43%) and *Vitis vinifera* (0.91%) [28,29,30,31]. NBS-LRR genes contain three domains: an N-terminal TIR/CC domain, a central NBS domain, and a C-terminal LRR domain [32,33]. The kiwifruit NBS-LRR genes can be divided into regular genes and non-regular genes. The regular NBS-LRR genes have all the three domains and complete open reading frames whereas the non-regular NBS-LRR genes lack at least one domain. In the kiwifruit genome, 20 CC-NBS-LRR genes and one TIR-NBS-LRR gene were detected and the other 79 genes were all non-regular NBS-LRR genes. These genes could be considered to be truncated or pseudogenes. Truncated NBS-LRR genes have been found in many plant genomes and they often surround regular NBS-LRR genes [34]. Non-regular NBS-LRR genes were inferred to arise through the partial deletion of redundant NBS-LRR genes. There was obvious difference between the numbers of CC-NBS-LRR genes and TIR-NBS-LRR genes. This phenomenon was also observed in other plant species especially dicots, such as *V. vinifera* (203 CNL and 97 TNL) and tomato (118 CNL and 18 TNL) [30,35]. In the Brassicaceae, Fabaceae, Solanaceae and Poaceae families, the TIR-NBS-LRR genes were more abundant [36]. There has been evidence suggesting that the CC-NBS-LRR genes may be the more ancient group than others in the phylogenetic tree of the NBS-LRR genes. Comparisons across plant species have also demonstrated a greater degree of diversity among CNL proteins than TNL proteins [37]. The greater number of CC-NBS-LRR genes suggested that the CC domain is more functionally active in its resistance to pathogens.

In this study, we found that 24 of the 63 NBS-LRR genes with accurate chromosome location information were distributed in nine gene clusters. Genes in the same cluster were commonly similar in sequence. Previous studies have revealed that NBS-LRR genes often cluster as tightly linked genes with high homology [38]. For instance, in cassava, seventy percent of the NBS-LRR genes are located within clusters [31]. In *Medicago truncatula*, approximately 309 NBS genes are located in 78 gene clusters [11]. The arrangement of the NBS-LRR genes with high homology in clusters possibly resulted from duplication, which includes whole genome duplication and tandem gene duplication. Previous studies found that the kiwifruit genome experienced several instances of polyploidization during its evolutionary process [39]. Hence, we speculated that the polyploidization-resulted whole genome duplication was one of the reasons leading to the formation of kiwifruit NBS-LRR gene clusters. After polyploidization, recombination and tandem gene duplication could also lead to cluster formation. A single functional gene locus is usually stable and under a low selection pressure [40]. For example, the R-gene *RPM1* that functions in resistance to *P. syringae* has only one copy and is conserved in *B. rapa* [41]. On the other hand, NBS-LRR genes located in clusters are polymorphic and evolve more rapidly. These genes in clusters, which are similar in sequence, provide possibilities for the formation of new variations [16,42]. As a result, the clustering, recombination and divergent selection of NBS-LRR genes caused the generation of novel resistance specificities to pathogens.

Cis-acting elements play roles in regulating transcriptional initiation and gene expression levels. The transcriptional initiation of resistance genes started through transcription factors recruited by cis-acting elements in promoters when plants were infected by pathogens. We screened the promoter sequences of the kiwifruit NBS-LRR genes and identified a large number of cis-acting elements belonging to 17 families. The distribution of these elements in promoters was not even and some specific sites tend to hold more elements than other sites. This distribution characteristic also appeared in other plants, such as blueberry, where cis-acting elements showed a positional bias toward the 900–800 nucleotides upstream from the transcriptional initiation site [43]. The genes with a close evolutionary relationship always showed similar element components, indicating they play similar roles in plant defense mechanisms. The AP2 transcription factors and ERF transcription factors belong to the AP2/ERF superfamily, which has been proven to participate in various resistance responses [44,45].In the study of *P. syringae,* which causes various diseases in crops including kiwifruit, the expression of specific AP2/ERF transcription factors enhanced disease resistance in tobacco and Arabidopsis, respectively [46,47,48]. Wurms et al. found that Psa infection caused an upregulation of AP2/ERF2 transcription factor in kiwifruit [49].

The expression of some specific resistance genes would be induced when the host was infected by corresponding pathogens. To analyze if these NBS-LRR genes play roles in kiwifruit defense mechanisms, we examined the expression pattern of 16 NBS-LRR genes after Psa infection. The results showed that expressions of three genes were significantly regulated upon Psa infection. The CNL-type gene *Achn112661* was significantly upregulated by Psa. A homolog of *Achn112661* in Arabidopsis encodes a resistance protein RPP13 which can recognize the causal agent *Hyaloperonospora arabidopsidis* (Hpa) [50,51]. Psa infection significantly downregulated the expression of *Ach337321,* which encodes the RPM1-like protein. This is consistent with our previous transcriptome sequencing result [52]. RPM1 was one of the resistance proteins studied thoroughly so far in plant immune responses. It recognizes the *P. syringae* avirulence protein AvrB in effect-triggered immunity and induces hypersensitive responses [26,27].

## 4. Materials and Methods

### 4.1. Identification and Classification of Kiwifruit NBS-LRR Genes

Genome sequence information including assembly and annotation was downloaded from kiwifruit (*A. chinensis* var. *chinensis*, Hongyang, 2n = 2x = 58) genome database (http://bioinfo.bti.cornell.edu/cgi-bin/kiwi/download.cgi). Predicted kiwifruit protein sequences were scanned for the Hidden Markov Model (HMM) profile of the NBS/NB-ARC domain (pfam00931) in HMMER v3.1b2 using “hmmsearch” with an e-value threshold of < 1e^-04^. The resulted candidates were then confirmed using NCBI conserved domain database (http://www.ncbi.nlm.nih.gov/Structure/cdd/wrpsb.cgi). The Pfam database (http://pfam.xfam.org/) was used to confirm the presence or absence of additional domains such as TIR and CC in the N-terminal position, and the variable number of LRR domains in the C-terminal position. The final confirmed NBS-LRR genes were classified based on their domain arrangement.

### 4.2. Phylogenetic Analysis, Gene Structure and Chromosome Localization

Multiple sequence alignments were conducted on the 100 kiwifruit NBS-LRR protein sequences using Clustal W [53] with default parameters. A neighbor-joining (NJ) phylogenetic tree was made using Clustal X [54] based on the NBS-LRR protein sequences with 1000 bootstrap replications and visualized with MEGA 5 [55]. The general feature format file of the kiwifruit genome was downloaded and used to retrieve the gene exon/intron structure. Chromosomal location information was retrieved from the results of kiwifruit genome assembly and annotation, and the distribution map was generated by Circos V0.69 [56]. The gene cluster was defined when it contained at least two genes and the distance between the two neighboring genes was < 200 kb.

### 4.3. Cis-Acting Element Analysis and Subcellular Location Prediction

In order to predict the cis-acting elements in the promoter regions of the kiwifruit NBS-LRR genes, we retrieved the 1500 bp sequence (from the ATG start codon) upstream of each gene. Cis-elements were predicted and analyzed using the PALCE database [57]. The subcellular localization was analyzed in silico using Plant-mPloc [58].

### 4.4. Inoculation of Psa and Quantitative RT-PCR Analysis

Kiwifruit cultivar “Hongyang” (*A. chinensis* var. *chinensis*) was used in this study. Twenty shoots were collected from 5 kiwifruit trees and stuck in MS medium and maintained in growth chamber. After 5 days, the stems were inoculated with Psa which caused the kiwifruit bacterial canker. Bacterial cells suspended in distilled water (OD600 = 0.2) was injected into the stems after they were carved with a knife. The control was inoculated with water. Phloem was collected after 48 h with three biological replicates.

Total RNA was extracted from the collected phloem according to the method of Cai [59]. All the 20 CNL-type genes and one TNL-type gene were chosen for qRT-PCR. Primers were designed with Primer5 software (Table S3) and *AdActin* was used as the internal control gene. The SYBR^®^ *Premix Ex TaqTM* (Perfect Real Time, Dalian, China) (TaKaRa Code: DRRO41A) was used for PCR, with conditions of 40 cycles of 95 °C for 20 s, 60 °C for 20 s, and 72 °C for 40 s. The relative gene expression was calculated according to the 2^−^^△△^*^C^*^t^ method [60].

## 5. Conclusions

In the last decade, kiwifruit has become more and more loved by consumers due to its unique flavor. However, with the growth of its planting area, various diseases are threatening the whole kiwifruit industry, such as the bacterial canker, which has hit the main kiwifruit planting countries heavily. As the biggest member of resistance genes in plants, NBS-LRR genes are considered to be the most important resistance genes. In order to discover possible resistance genes, we made use of the genome sequence of kiwifruit for the identification and characterization of NBS-LRR genes. In the present study, we identify 100 NBS-LRR genes in the kiwifruit genome and they were grouped into six distinct classes based on their domain architecture. These NBS-LRR genes are distributed unevenly across 18 kiwifruit chromosomes and 38.01% of them are present in clusters. Seventeen families of cis-acting elements were identified in the promoters of the NBS-LRR genes, including AP2, NAC, ERF and MYB. Psa infection induced the differential expression of 16 detected NBS-LRR genes and three of them are involved in plant immunity responses. Our study provides an insight into the NBS-LRR genes in kiwifruit and a resource for the identification of new *R-genes* in kiwifruit.

## Figures and Tables

**Table 1:** Classification of the Nucleotide-binding site and leucine-rich repeat (NBS-LRR) genes in the kiwifruit genome.

Class	Type	Number of Genes
1	CC-NBS	3
2	CC-NBS-LRR	20
3	TIR-NBS	1
4	TIR-NBS-LRR	1
5	NBS	20
6	NBS-LRR	55
Total	Total	100

## Supplementary Materials

Supplementary Materials can be found at https://www.mdpi.com/2223-7747/9/10/1350/s1. Figure S1:Cis-acting elements of promoters of the NBS-LRR genes, Table S1: Data of the 100 NBS-LRR protein coding sequences, Table S2: Subcellular location prediction of the NBS-LRR genes, Table S3: List of primers used in qRT-PCR.
