# Supplementary material for: Identification and Analysis of NBS-LRR Genes in Actinidia chinensis Genome
Source: Plants (Basel). 2020 Oct 13;9(10):1350. doi: 10.3390/plants9101350 (PMC7601643; doi:10.3390/plants9101350)
Supplement: Supplementary file 1 [file plants-09-01350-s001.zip › supplementary materials/Table S3 List of primers used in qRT-PCR.docx]

Table S3 List of primers used in qRT-PCR

| Gene | Sense primer (5'→3') | Anti-sense primer (5'→3') |
| --- | --- | --- |
| Achn237541 | CAACAAACCCTAACTGCAGGAIAGT | ACCITCCCCGAATCACCCTTCCTTG |
| Achn334411 | ATGGCCGACGACACCACCTGCTTCT | AAAAACACCACCAATCATCCGCCAT |
| Achn334451 | ATGACCACCGTCAAAACCCAAGAAG | CCTCCTGCCACTTCACCGTCGTCAT |
| Achn369901 | ATGACCCGCTGCACCTACCAAGTCA | ACACCTGATACCAACACCGCCACAT |
| Achn369911 | ACCGTCGGCGAAGCCTTCGAAAAAC | GCTTCTCAAACGCCTCCCCCACCCA |
| Achn369921 | ATGGCCGCCGTCCGCGCCCAAGAAG | CCTCCTGCGCCCGCACCGCCGCCAT |
| Achn370391 | ATGGCCGCCTTCTTCCCCCCCCCCA | ACGGCGGCGGAAAAAACGCCGCCAT |
| Achn178051 | ATGGCCGAAAACGACACCAAACCCG | CCGGCTTCCAATCATTCTCCGCCAT |
| Achn066161 | ATGGCCGGCGACGGCGACGACGAAT | ACTCATCATCCCCATCCCCCGCCAT |
| Achn096341 | ATGAAAGAAACCGACGAACGCAAAC | GCTTCCGCTCATCCGTCTCCTTCAT |
| Achn112411 | ATGGCCGGCCAAGAAGCCACCACCA | ACCACCACGCCTCCTGCCCCGCCAT |
| Achn112661 | ATGGCCGCCGTCCGCGCCCAAGAAG | CCTCCTGCGCCCGCACCGCCGCCAT |
| Achn228931 | ATGGGCGACCCCATTGCCCTCGTCG | CCACCAACGCAATCGGATCCCCCAT |
| Achn337321 | ATGGCCGAAGCCGCCGTCATTAACC | AATTAATCACCGCCGCCTCCGCCAT |
| Achn340061 | ATGGCAATGATCGCTGTCAATGTCG | CAACTTTCACTGCAATCATAGCCAT |
| Achn208841 | ATGGCCGAAAACGACACCAAACCCG | CCGGCTTCCAATCATTCTCCGCCAT |
